# 3,5-T2 and 3,3′,5-T3 Regulate Cerebellar Thyroid Hormone Signalling and Myelin Molecular Dynamics in Tilapia

**DOI:** 10.1038/s41598-019-43701-w

**Published:** 2019-05-14

**Authors:** Y. Hernández-Linares, A. Olvera, P. Villalobos, C. Lozano-Flores, A. Varela-Echavarría, M. Luna, A. Orozco

**Affiliations:** Instituto de Neurobiología, Universidad Nacional Autónoma de México (UNAM), Querétaro, QC Mexico

**Keywords:** Developmental biology, Molecular biology, Neuroscience

## Abstract

In contrast to mammalian adults, myelination in teleosts occurs throughout their lifespan and most of the progenitor cells are originated in the cerebellum. To understand the role that thyroid hormones (THs) play in juvenile cerebellar myelination in teleosts, we identified and localised the expression of genes involved in TH signalling (mct8, oatp1c1, dio2, dio3, thraa and l-thrb1) and analysed the effects of the two bioactive THs, T2 and T3, upon their regulation, as well as upon some structural components of the myelination process. *Ex vivo* approaches using organotypic cerebellar cultures followed by FISH and qPCR showed gene-specific localisation and regulation of TH signalling genes in the cerebellar nuclei. *In vivo* approaches using methimazole (MMI)-treated juvenile tilapias replaced with low doses of T3 and T2 showed by immunofluorescence that myelin fibres in the cerebellum are more abundant in the granular layer and that their visible size is reduced after MMI treatment but partially restored with TH replacement, suggesting that low doses of TH promote the re-myelination process in an altered condition. Together, our data support the idea that T2 and T3 promote myelination via different pathways and prompt T2 as a target for further analysis as a promising therapy for hypomyelination.

## Introduction

Thyroid hormones (THs) are key endocrine messengers that exert their actions mainly by binding to their nuclear receptors (TRs) to regulate gene expression. To this end, three functional events need to occur: (1) TH transport to the target cell, mediated by two proteins, the organic anion transporter polypeptide type C1 (OATP1C1) and the monocarboxylate transporter type 8 (MCT8)^1–3^; (2) the tissue-specific activation/inactivation of the prohormone thyroxine (T4) to either the bioactive 3,3′,5-triiodothyronine (T3) or the inactive rT3 catalysed by deiodinases D2 and D3, respectively^4^; and (3) the binding of T3 to the TR to regulate transcription of target genes^5^. Aside from T3, 3,5-diiodothyronine (T2), a by-product of T3 outer-ring deiodination, is another relevant transcriptional modulator^6–9^. Indeed, T2 has been shown to bind to and activate at least both the human TRβ1 and a long isoform of a teleostean TRβ1 (L-TRβ1), which contains a 9 amino acid insert within the ligand binding domain that is primarily activated by T2^6–9^.

THs are crucial for myelination in all vertebrates^10^. Development, establishment and maintenance of myelin cytoarchitecture require a tight modulation of TH, and slight defects in the processes could affect the entire system, leading to severe neuropathy^11^. In fact, THs and other thyroid axis components like TRH have been proposed as promising treatments for pathologies, such as multiple sclerosis, due to their effects upon remyelination^12,13^. The cerebellum is a well-established centre of control and coordination of movements. THs are also key for mammalian cerebellar development, where perinatal hypothyroidism severely affects oligodendrocyte (OL) differentiation and myelination, synaptic connections among cerebellar neurons and the density of afferent neuronal fibres^14,15^. Myelination in more advanced stages of vertebrate ontogeny have been poorly explored, partly because the oligodendrocyte progenitor cells (OPCs) in adults are highly restricted to certain proliferative zones, like the subventricular zone (SVZ) and subgranular zone (SGZ)^16^, as well as the cerebellum. In contrast, teleosts exhibit several proliferative zones within the central nervous system (CNS) throughout their lifespan^17^ and the localisation of stem cell niches is conserved among different species, with most progenitor cells originating from the cerebellum^18,19^. Teleost continuous neurogenesis is explained by the persistence of the radial glia beyond early development^20^. In particular, during the juvenile stage, a window of action for TH agonists and antagonists has been shown to compromise cerebellar physiological processes (connections, dendritic arborization, persistent myelination, among others)^15^. Thus, the juvenile teleost cerebellum is an excellent model system to study TH actions upon myelination processes in non-developing vertebrates^20^.

The mechanisms of TH regulation in the cerebellum remain unclear; however, we recently described two different gene populations associated with myelination and development regulated either by T2 or T3 in juvenile tilapia cerebellum, suggesting that these two hormones could elicit specific and non-redundant functions in this tissue^9^. As an initial approach to understand the role that THs play in juvenile cerebellar myelination in teleosts, in the present work we identified and localised the expression of genes involved in TH signalling and analysed the effects of the two bioactive THs, T2 and T3, upon their regulation. In parallel, we explored the regulatory effect of these hormones on some structural components of the myelination process.

## Results

### T3 and T2 regulate the expression of genes associated with thyroid function in the cerebellum

To investigate if the three sets of genes involved in TH action (transporters, deiodinases and receptors) were expressed in the teleost cerebellum, as well as their possible regulation by THs, organotypic cerebellum cultures were exposed to different T2 and T3 concentrations, and RT-qPCR analysis was used to quantify gene expression. Both TH transporters were expressed and down-regulated by THs (Supplementary Fig. S1a). However, while mct8 expression was repressed in all TH concentrations tested, oatp1c1 down-regulation was significant at high T2 concentrations. Surprisingly, dio3 expression was not regulated by any treatment, but dio2 was down-regulated by both T2 and T3 (Supplementary Fig. S1b). As previously observed, l-thrb1 was only up-regulated by T2, while thraa was down-regulate by both hormones (Supplementary Fig. S1c). With these results we identified that the exposure of cerebellar organotypic cultures to 10 nM of either hormone elicited significant changes in the regulation of the analysed genes.

### Expression of genes associated with thyroid function are differentially localised in the cerebellum

**Figure 1:**
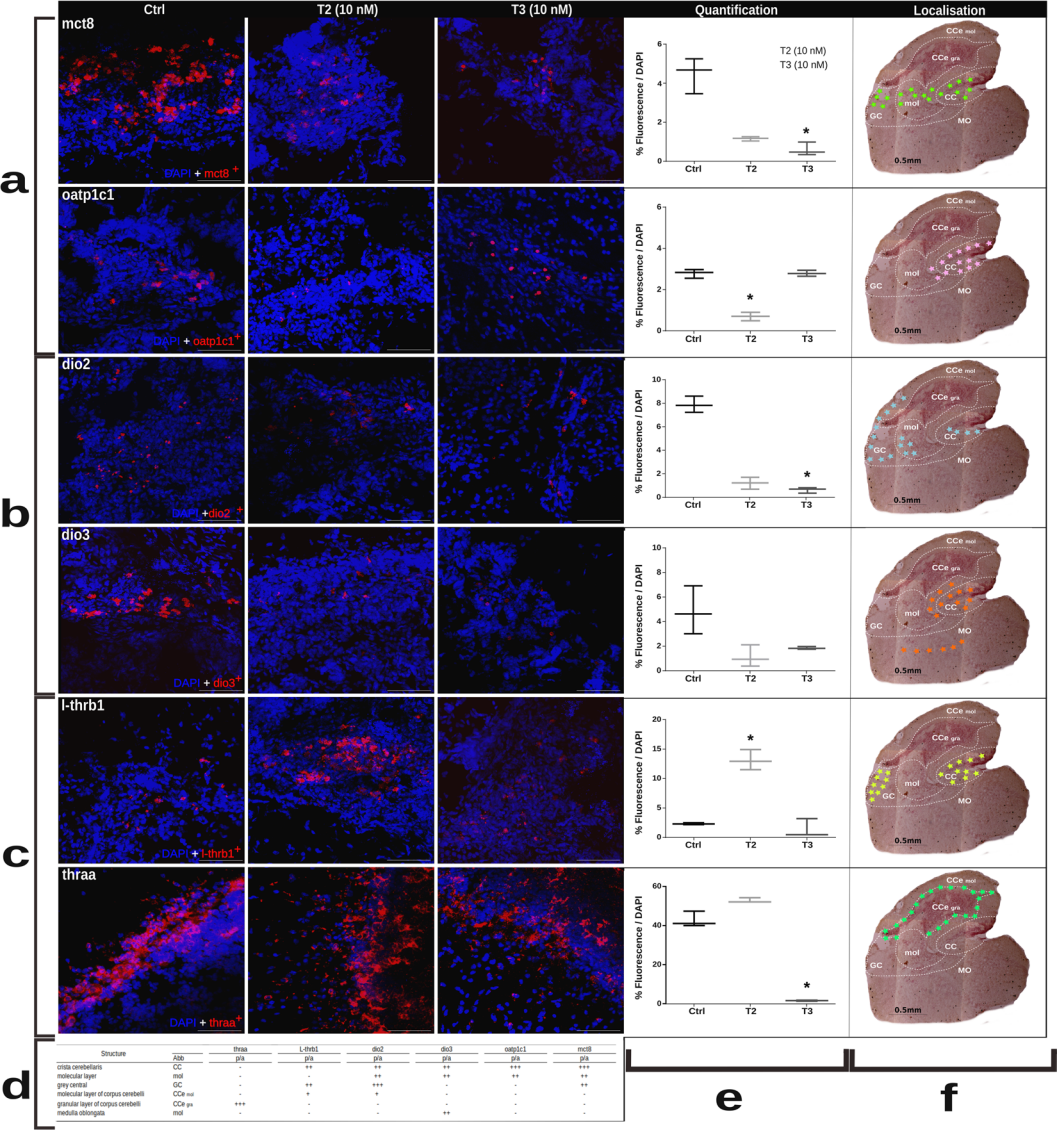
Fluorescent *in situ* hybridisation analysis. *Ex vivo* experiments for the quantification of mRNA expression in confocal images of sagittal sections of cerebellum in control and treated groups show the localisation of the probes for each gene. mRNA expression of the different genes was detected with Cy3 in red and the signal of DAPI in blue. The scale bar represented 50 μm. (**a**) Transporters of THs, (**b**) deiodinases, (**c**) receptors of THs (**d**) table showing the abundance [low (+), medium (++) or high (+++)] or absence (−) of expression in each structure that conforms the cerebellum. **e**) Quantification of total fluorescence normalised with DAPI. For all graphs * is p < 0.05 and (**f**). Photomicrographs of the same sections with Nissl staining showing in pointed lines the definition of the different nuclei that comprise the tilapia cerebellum. The zone of expression of each gene in the control groups is marked in colour stars.

Slices of cerebella exposed to 10 nM T2 and T3 were analysed by using the FISH technique to identify the expression of all three sets involved in TH action. Confocal images of sagittal sections allowed the identification of cerebellar nuclei-specific expression of the different genes. Results are shown in Fig. 1 and Supplementary Fig. S2. mct8 is expressed in the grey central and co-localises with oatp1c1 in the crista cerebellaris and molecular layer (Fig. 1a,f). Both deiodinases (dio2 and dio3) are expressed in the crista cerebellaris; additionally, dio2 expression was identified in the grey central and dio3 in the molecular layer and medulla oblongata (Fig. 1b,f). TR genes did not co-localise; l-thrb1 was expressed in grey central, molecular layer and crista cerebellaris, whereas thraa was abundantly expressed in the peripheral granular layer of the corpus cerebelli, where Purkinje cells are located (Fig. 1c,f). The analysis of the expression of the different genes after T3 or T2 treatments by FISH showed the same regulatory pattern as observed in the analysis by RT-qPCR (Fig. 1e). However, FISH allowed the observation of TH regulatory response after an acute treatment.

### T3 and T2 regulate the expression of genes related to myelination in the cerebellum

**Figure 2:**
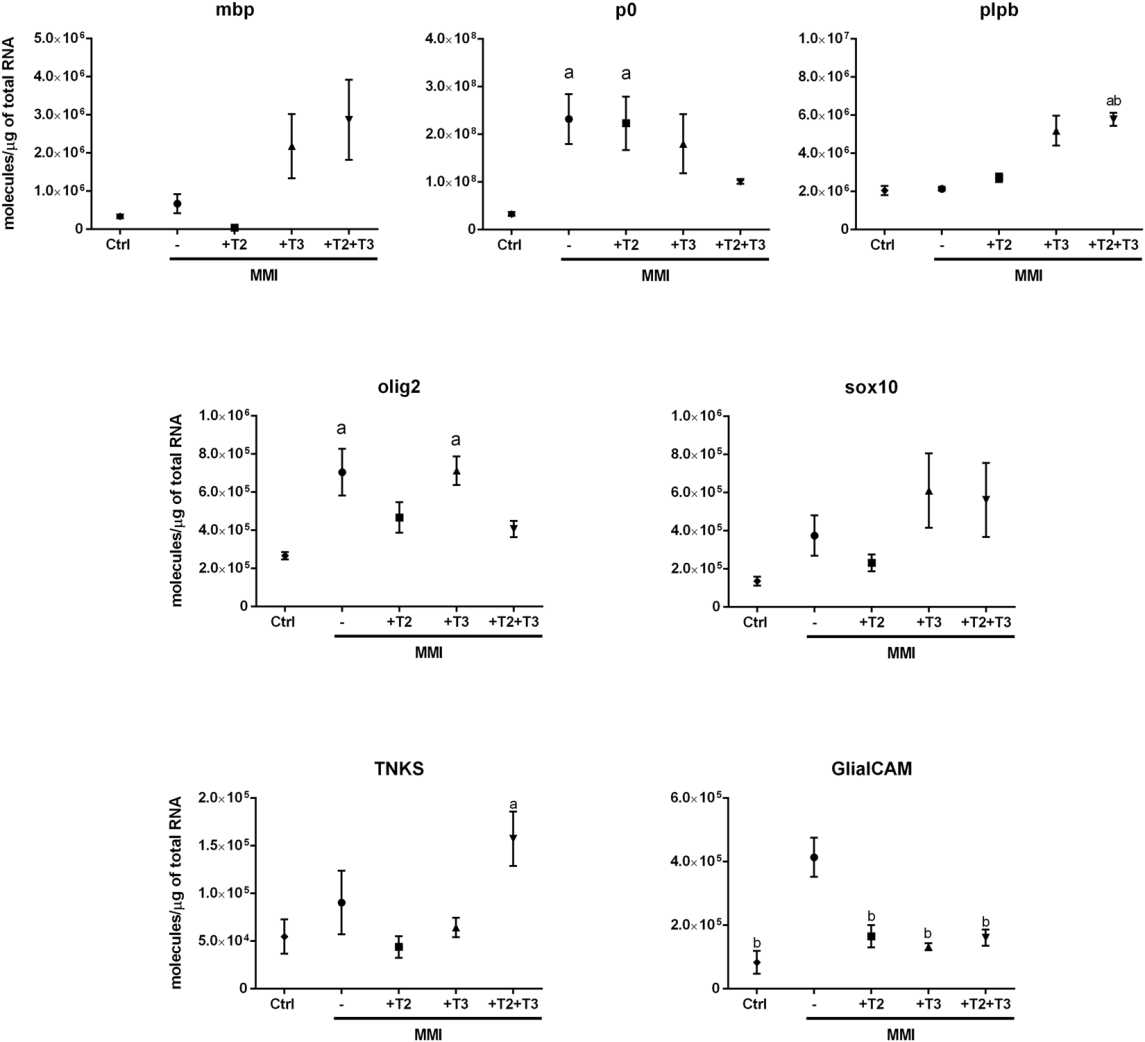
Cerebellar mRNA expression of mbp, p0, plp1b, olig2, sox10 tnks and GlialCAM. Tilapia were exposed to 4.5 mM MMI with or without simultaneous addition of 1 nM T2, T3 or T2 + T3 for 30 days. Values are means +/− S.E.M. Significance is indicated p < 0.05 with respect to control group.

To assess the involvement of T3 and T2 in cerebellar myelination, we went back to the *in vivo* model and treated juvenile tilapias with MMI to partially block TH synthesis with or without co-administration of T2, T3 or a combination of T2 + T3 in sub-physiological and equimolar doses (1 nM) for 30 days. In contrast to the observations in cerebellar organotypic cultures (Supplementary Fig. S1), genes involved in TH signalling in the *in vivo* experiments were not significantly altered after 1 nM of TH treatment for one month (Supplementary Fig. S3). However, the expression of the genes olig2 and sox10, as well as mbp, p0 and plp1b, described as oligodendrocyte precursor cells (OPCs) and mature oligodendrocyte markers, respectively^21^, was modulated in a TH-specific manner by T3 and T2 (Fig. 2). Cerebellar expression of plp1b was up-regulated by T2 + T3; p0 was up-regulated after MMI and MMI + T2, suggesting that only T3 restored control expression of this gene; T2 restored mbp expression when compared to MMI-treated groups; sox10 expression was up-regulated by T3, and only T2 restored control expression of olig2 after MMI treatment.

Two genes that participate in the mammalian myelination process were previously identified in the tilapia cerebellum transcriptome: tankyrase (tnks) and GlialCAM. These genes were differentially regulated by T2 and T3, respectively^9^. As seen in Fig. 2, under the experimental conditions used for the present work, GlialCAM expression was up-regulated after MMI treatment, and co-administration of T2, T3 or T2 + T3 restored mRNA levels to those of non-treated controls. tnks expression, however, was up-regulated only in the hypothyroid group co-treated with T2 + T3.

### Thyroid status alters the diameter of myelin fibres in the cerebellum

**Figure 3:**
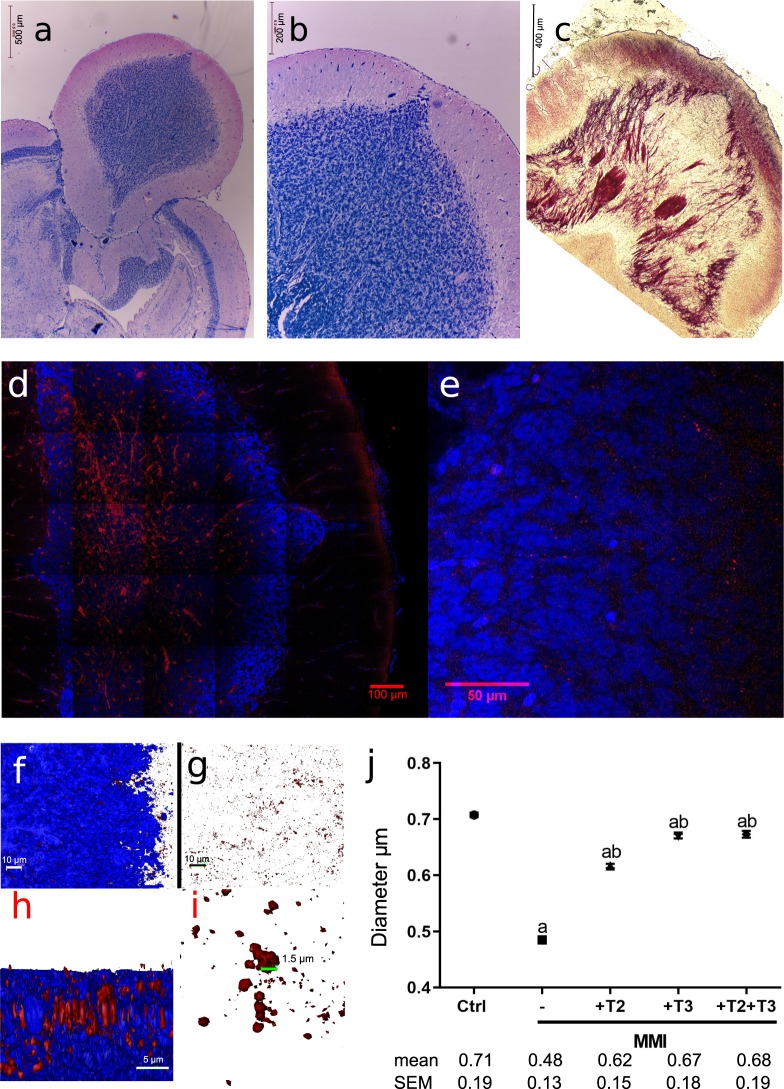
Myelin distribution and composition in tilapia cerebella. (**a**,**b**) Coronal sections of cerebella (5 μm) in Kluver-Barrera staining. (**c**) Coronal sections of cerebella (50 μm) in black-gold staining. Bundles of myelin fibres pass through the granular layer. (**d**,**e**) Confocal microscopy of 20 μm coronal sections of the cerebellum of a control animal, immunostained to myelin basic protein (red) and counterstained with DAPI (blue). Note that stained myelin proteins and fibres are more abundant in the granular layer. (**f**) Frontal view of the reconstruction of 30 confocal sections showing the merge of red for myelin and blue for DAPI. (**g**) Frontal view of 30 confocal sections for a 3D reconstruction showing only the red channel for myelin. (**h**) Lateral view of a 3D reconstruction showing myelin fibres. (**i**) Zoom of myelin fibres in a frontal view. 3D reconstruction and measurement of myelin fibre diameters were performed with Amira software. A total of 9 images (3D reconstructions) within the granular layer from 3 different cerebella per group were analysed. (**j**) Myelin fibre apparent diameters of tilapia exposed to 4.5 mM MMI with or without simultaneous addition of 1 nM T2, T3 or T2 + T3 for 30 days. Values are means +/− S.E.M of ~700 single measurements per group. Significance is indicated as *p* = 0.001 compared to the control (**a**) or MMI-treated group (**b**) ared to the control (**a**) or MMI-treated group (**b**).

The tilapia cerebellum consists of 3 major layers: the granular layer, the Purkinje cell layer and the molecular layer, resembling a single folium of the convoluted mammalian cerebellum (Fig. 3a). As seen through two distinct myelin staining techniques (Fig. 3b,c), and further confirmed by immunofluorescence (Fig. 3d), myelin fibres are more abundant in the granular layer, where cell density is also higher. We measured myelin fibre diameters in the granular layer in order to analyse myelination in response to thyroid status (Fig. 3e–j). Myelin fibre diameters were reduced after one month of MMI treatment and partially restored with TH replacement, suggesting that TH treatment in low doses promotes re-myelination in an altered condition (Fig. 3j; Supplementary Video S7).

## Discussion

TH actions and effects during neurodevelopment are well known and conserved across different vertebrates; however, little is known about their role in adult neurogenesis^22^. In teleosts, a substantial percentage of mitotic cells within the proliferative zone are located in the cerebellum, and this proportion is maintained beyond early development and throughout adult life^16,18^. Indeed, a clear proliferative zone has been identified in the teleostean cerebellum, preserving this structure the ability to generate and regenerate neurons and glial cells in juvenile and adult stages^16,18^. In the present study, we questioned the effects of the two bioactive THs, T3 and T2, on cerebellar TH signalling, and their participation in juvenile myelination.

As far as we know, this work shows for the first time cerebellar mRNA expression as well as T3 and T2 regulation of TH transporters oatp1c1 and mct8; deiodinases dio2 and dio3, and TH receptors thraa and l-thrb1, supporting the involvement of these genes in cerebellar TH signalling. These genes showed a differential distribution, and all except thraa were prominently expressed in the crista cerebellaris, probably because of its strategic localisation as a transition nucleus between the medulla oblongata and corpus cerebelli, an important passageway for neuronal afferences and vascular irrigation^21,23,24^.

Genes regulated by the acute exposure to T3 or T2 showed similar mRNA expression patterns with both qPCR and FISH. Cerebellar mct8 was more highly expressed than oatp1c1; this can be explained by the broader MCT8 cell type distribution (neurons, oligodendrocytes, precursors and endothelial cells), as compared to OATP1C1, which is only observed in endothelial cells in fish^25^. The down-regulation exerted by THs on their transporters may be due to a negative feedback when sensing an increase in circulating hormones. Notably, we found that oatp1c1 was only regulated by T2, allowing the hypothesis that this hormone could be a possible substrate for this transporter. Knock-down and knock-out models in mammals, birds and zebrafish have shown that mct8 and oatp1c1 expression and protein location are essential for proper neuronal and glial differentiation and migration to establish the cerebellar layers^26,27^. mct8 and oatp1c1 transporters were identified in the crista cerebellaris and molecular nucleus located in the ventral-posterior cerebellar zone, and they are probably key for the systemic arrival and transit of hormones to the CNS through the blood-brain-barrier. Indeed, cerebellar irrigation in teleosts depends on the central arteries passing through the ventral-posterior zone^21,23^.

The co-localisation of mct8 and oatp1c1 with both types of deiodinases agrees with the idea of the fine tuning of the TH signalling pathway in which intracellular TH transport, followed by TH activation/inactivation, is crucial for the balance of TH bioavailability. Contrary to previous reports on the liver, we showed that cerebellar dio3 was not up-regulated by T2 or T3, indicating tissue-specific regulation within the CNS. dio2, however, was down-regulated by T2 and T3, as reported for all other tissues^7^, showing its fundamental role as a bioactive TH gatekeeper.

Interestingly, present results showed that cerebellar expression of l-thrb1 and thraa did not co-localise. thraa expression was detected only in the periphery of the granular layer, where Purkinje and eurydendroid cells are localised. These cell types project to the molecular layer and to different nuclei within and beyond the cerebellum^24^. The possible functional role of thraa in this cerebellar layer has yet to be explored. In contrast, l-thrb1 expression was detected in the crista cerebellaris and grey central, co-localising with all other analysed thyroid signalling-related genes (mct8, oatp1c1, dio2 and dio3), suggesting a clear TH-dependent function in these cerebellar structures. Present results contrast with those previously reported in zebrafish^28^, which showed a high distribution of thraa and a restricted localisation of thrb to certain nuclei. The observed differences in the specific localisation and levels of expression of the analysed genes could reflect a dynamic process associated with particular physiological scenarios. However, data in zebrafish and the results of this study showing differential localisation and TH regulation support the idea that thraa and thrb exert different roles in the CNS, possibly regulating different biological functions. In this context, in mammals, TRα has been associated with CNS development and neurogenesis^28^, whereas cerebellar TRβ has been involved in myelination through the regulation of Krüppel-like factor 9 (KLF9) in differentiating oligodendrocytes^13^.

To further analyse the involvement of THs in the juvenile myelination process, TH synthesis was blocked by means of a long-term (1 month) intermittent exposure to MMI either alone or with a low (1 nM) TH replacement. This MMI administration protocol has been shown to drop T3 intrahepatic concentrations to around 50%, as compared with controls when administered alone^7^. Unexpectedly, no significant differences were detected in the regulation of genes related to TH signalling in the tilapia cerebellum; however, the expression of this same set of genes does respond to both MMI alone or co-administered with THs (data not shown), suggesting the instalment of a compensatory mechanism. In contrast, modulation in the expression of OPCs and mature oligodendrocyte markers was observed, suggesting that the possible discrete TH fluctuations resultant from the intermittent and low dose administration protocol could influence the molecular dynamics of oligodendrocytes. In the rodent cerebellum, the expression of early OPC Olig-1 or OPC PDGFαR markers is prominent in the perinatal period and decreases gradually as development progresses (between 3 and 14 postnatal days, when the expression of mbp, a mature oligodendrocyte marker, increases). This switch is maintained throughout adulthood, where neurogenesis is restricted^29^. In juvenile tilapia, however, we observed no clear switch between OPCs and mature oligodendrocyte markers since both are expressed at similar levels. This can be explained by the high neurogenic potential maintained throughout teleost lifespan, where the majority of adult-born cells are originated in the cerebellum^20^. The fact that the expression of OPCs and mature oligodendrocyte markers was modulated by THs in juvenile cerebellum suggests that this endocrine signalling pathway is relevant for juvenile-adult myelination. Another signalling pathway relevant for mammalian differentiating oligodendrocytes is the Wnt signalling pathway, which under physiological conditions is repressed by TNKS for proper myelination^30^. Furthermore, the glial cell adhesion molecule, or GlialCAM, is a protein involved in oligodendrocyte myelination in mice^31^. T2 and T3 could be modulating myelination through tnks and GlialCAM mRNA regulation as seen in PCR and transcriptomic experiments. To explore this, we took advantage of the mild hypothyroid state resulting from the MMI intermittent treatment^7^ and measured the apparent diameter of myelin fibres located in the granular layer of the cerebellum by immunofluorescence. The decrease in TH availability resulted in a significant reduction of myelin fibres (Fig. 3j). Interestingly, replacement with low doses of T3, T2 and T3 + T2 resulted in a significant recovery of myelin fibre diameters, showing that T3 and T2 may regulate myelin formation in juvenile tilapia cerebellum. Further studies using more potent visualization methodologies like electron microscopy will be useful to confirm these data. Although it is not clear if OPCs constitutively differentiate into myelin forming cells in mammals^32^, it has been shown that T3 improves the remyelination process in a juvenile demyelination rodent model, functioning as a neuroprotective agent by reducing proliferating cells in the SVZ and promoting OPC maturation^29,33^. Furthermore, TH analogues have been tested as promoters of re-myelination in an effort to ameliorate the symptoms of the human CNS-localised hypothyroidism seen in Allan-Herndon-Dudley syndrome (AHDS). This disorder results from a mutation in the *mct8* gene that inhibits T3 entrance to the brain, leading to demyelination with severe psychomotor retardation. Some of the most promising TH analogues are diiodothyropropionic acid (DITPA) and triiodothyroacetic acid (TRIAC). In this context, DITPA had an effect on the expression of brain TH-signalling genes of *mct8* knock-out mice only if treated prenatally^34^. Furthermore, human oligodendrocytes treated with DITPA up-regulated the expression of genes related to oligodendrocyte differentiation and myelination, and oligodendrocyte co-culture with retinal ganglion axons promotes myelination after DIPTA treatment^35^. In teleosts, the only model for hypomyelination is the mct8-/- zebrafish larvae, which resembles AHDS. Hypomyelination was partially or totally rescued with a 3-day treatment of DITPA or TRIAC, respectively^27^. The fact that a T2 analogue had positive effects upon myelination suggests that T2 could also ameliorate myelination in different vertebrates. In the present study, l-thrb1 expression was up-regulated by T2 in the crista cerebellaris (Fig. 1), a nucleus with transit of ascendant tracts to the granular layer, suggesting that T2 via its receptor could participate as a signal in myelination regulation. Additionally, T2 action is independent of MCT8, like TRIAC and DIPTA^36^, compounds that have demonstrated CNS myelination therapeutic potential in case of mutations or total deletion of MCT8^27^. The fact that T2 down-regulates cerebellar mct8 mRNA could imply T2 participation in other CNS processes. Altogether, these studies and our data support the idea that T2 and T3 promote myelination via different pathways, and prompt T2 as a promising target to analyse for therapy on hypomyelination.

## Materials and Methods

### Biological samples

Male tilapia juveniles (*Oreochromis niloticus*) ~4–6 g (16 weeks post-hatching) were kindly provided by the Quarantine Unit for Tilapia and Catfish at the Universidad Michoacana de San Nicolas de Hidalgo, Mexico. Fish were kept in 10 L tanks with aerated freshwater at a temperature of 25 °C on a 12:12 h photoperiod. Fish were fed once a day with a commercial diet (Sera Marin, Sera, Germany). All animal experimentation was conducted in accordance with accepted standards of humane animal care, and protocols and procedures regarding handling and euthanasia were reviewed and approved by the Animal Welfare Committee of our Institute.

### *Ex vivo* experimental design

We used an *ex vivo* tilapia cerebellar organotypic culture to analyse the acute effect of TH treatment upon the regulation of genes participating in TH signalling and myelination. mRNA expression was measured by both qPCR and FISH. Cerebellar organotypic culture protocol was performed as previously described for tilapia liver^7^, with the following modifications: Intact cerebella from non-treated tilapia were dissected and placed into ice-cold stabilizing medium. For each treatment, four cerebella were placed onto inserts of semi-porous membranes (pore size 0.4 μm) in 6-well culture plates containing culture medium and maintained in an incubator at 18 °C in 5% CO_2_. After 48 h of stabilisation, the culture medium was supplemented with vehicle **NaOH** (0.01 N) (control groups) or **T3** or **T2** at concentrations of (0.1, 1, 10, and 100 nM) previously solubilised in vehicle (experimental groups), and tissues were incubated for 24 h. Experiments for qPCR were performed in triplicate (n = 60 cerebella; 12 cerebella per each of 5 experimental groups) (Supplementary Fig. S1). For FISH experiments, a total of 12 cerebella were analysed (n = 4 cerebella/treatment), which included controls and two treated groups (10 nM of T2 and T3).

### *In vivo* experimental design

To analyse the effect of **T2** or **T3** on myelination *in vivo*, tilapia groups were treated with **methimazole** (MMI) (4.5 mM) (Sigma), a TH synthesis inhibitor, with or without simultaneous addition of 1 nM T2, T3, or T2 + T3 (Sigma) previously solubilised in (0.01 NaOH). Treatments were administered using an immersion protocol previously described and validated in our laboratory^7^. Briefly, MMI or MMI + TH was added to the culture water in the corresponding tank at 09:00 h and replaced with fresh culture water at 17:00 h. This drug administration protocol was repeated three times per week for 30 days. At the end of the experiment, animals were euthanised and cerebella were dissected and quick frozen in **TRIzol** (Invitrogen) at −80 °C until RNA extraction. Using the MMI administration protocol, T3 intrahepatic concentrations show a half reduction as compared to untreated tilapia. Treatment with 1 nM of T3 or T2 alone modifies the expression of some TH-dependent genes, showing that the organism is able to sense the slight fluctuations in intra-tissular TH concentrations^7^. Due to the intermittent nature of the protocol, treated fish were also exposed to discrete TH fluctuations receiving a boost of THs 3 times per week. For RT-qPCR, 3 cerebella were pooled to obtain 3 samples in each treatment. For immunofluorescence and histology analyses, the entire brains were fixed with **paraformaldehyde** (4%) for 3 h and 3 cerebella were analysed for each treatment.

### RNA extraction

Total RNA was extracted from tilapia cerebella collected from the *in vivo* experiments and the organotypic cultures with TRIzol. RNA purity and integrity were assessed by (1%) **agarose** gels and concentration was measured with a Nano-Drop ND-1000 UV-Vis spectrophotometer at 260 nm (Nano Drop Technologies). For each treatment, ~50–70 mg of tissue was used for total RNA extraction. RNase-free DNAse I (Invitrogen) was added to remove residual genomic DNA.

### RT-qPCR

Quantification of the different gene transcripts was performed as previously described^7^ Briefly, mRNA was reverse transcribed (RT) from (2 µg) of total RNA using an oligo(dT) primer. Quantitative PCR was carried out in duplicates using β-Actin and ubiquity-conjugating enzyme E2Z (UBCE) as reference genes^37^. PCR protocols and oligonucleotides used for gene amplification are specified in Supplementary Table 1S4. In all cases, gene-specific standard curves were used and in quantification and data analyses performed in a Step One instrument according to the instructions from the manufacturer (Applied Biosystems®). The protocol for all genes was 10″ at 95 °C, 10″ at 60 °C, 10″ at 72 °C for 40 cycles. For each experimental sample, the mRNA concentration was expressed as molecules per microgram of total mRNA used in the RT reaction (2 µg), obtained by comparison with the standard curve and normalised to the concentration of each reference gene.

### NISSL staining

Brains previously fixed in **PFA** (4%), cryopreserved in sucrose and paraffined, were sliced in 10 μm sagittal sections. Sections were gradually rehydrated with **xylene** and (100%, 96%, 80% and 70%) **ethanol** for 5 min each and dyed with (0.5%) **Cresyl Violet** solution for 30 sec, washed in distillate water and dehydrated with reverse series of alcohol solutions and xylene for 10 min to finally apply **Entellan** mounting medium.

### Fluorescent *in situ* hybridization (FISH)

After treatments (see above), cerebella were frozen in **2-methylbutane** (submerged in mix of dry ice/ethanol), and then embedded in Tissue-Tek O.C.T. Compound (Sakura Finetek^®^). The samples were sliced in a cryostat (Leica-CM1850) to obtain sagittal sections of 30 μm and mounted in SuperFrost^®^ Plus (VWR) slides. Sagittal sections allow not only the location of gene expression at the histological level but also the identification of tracts where they may have an action in the cerebellar system.

#### Synthesis of mRNA probes

The full coding region of TRα1, D2 and D3, as well as a 1 kb fragment of the coding region of L-TRβ1, OATP1C1 and MCT8 were amplified using specific oligonucleotides (Supplementary Table 2S5). Amplified fragments were inserted into a pCR^®^4-TOPO^®^ vector (Invitrogen) and linearised using Not1 and Spe1 or Pme1 restriction enzyme. All mRNA probes were labelled by *in vitro* transcription using T3 (antisense) and T7 (sense) RNA polymerase with **digoxigenin-UTP** DIG RNA labelling kit (ROCHE). Supplementary Fig. S6 shows sense and antisense controls.

#### Hybridisation

Tissue slices were treated as previously described by Carasatorre *et al*.^38^. Anti-**DIG-POD** (1:800) (ROCHE) diluted in **TSA** was added and the slices were incubated overnight at 4 °C, washed in **TBS** and **TTBS** and developed with Tyramide signal amplification-Cy3 (TSA-Cy3; 1:75) detection kit (Perkin-Elmer) in darkness and incubated for 60 min, washed in **TBS** and **TTBS**, contrasting with **DAPI** (1:400 in TBS) (ROCHE), and mounted with medium **1**,**4-Diazabicyclo[2**.**2**.**2]octane** (DABCO 33-LV; Sigma-Aldrich).

#### Fluorescent images

Observations and image acquisition were performed in a microscope Imager ZI (ZEISS) using AxioCam MRm (ZEISS- 60N-C). The images were obtained in 2D mosaics of 12 × 14 scan, saved in.jpg format in AxioVision LE 4.8.1 software 2009, and were size adjusted, cropped, contrast enhanced, and annotated in Inkscape (Vector Graphics Editor).

#### Confocal images

Cy3 (AB-514; EM-570) fluorescence was detected using confocal microscope Carl Zeiss – LSM 780 and ZEN software. Coherent-XR multiphoton laser at 350 nm was used for DAPI. The 3D images were obtained in Tile Scan from 10 to 18 µm of depth in Z (1.58 pixel), with 1024 × 1024 pixels of resolution, and analysed with the image-processing package of Fiji (version: 2.0.0-rc-66/1.52b; http://imagej.net/contributors) of the Image J program.

#### Total fluorescence

Total fluorescence intensity was calculated with the following formula (int.den. of red or blue channel) - (the total area * average background). Values of the integrated density were obtained using the Fiji toolbar of each separate channel [red (TSA-Cy3) and blue (DAPI)]. Total fluorescence was obtained as the percentage of TSA-Cy3 with respect to DAPI.

### Black-Gold II

Coronal sections were used for Black-Gold II, immunofluorescence and Kluver-Barrera staining to have the same orientation of the cerebella for myelin filament co-localisation and diameter measurement. For Black-Gold II, a modified protocol described by Schmued *et al*.^39^ was followed. Briefly, coronal sections of 50 μm were re-hydrated with **PBS** for 2 min at RT, and the slides were incubated in **Black-Gold II** solution at 60 °C for 20 min until the staining was complete, followed by sequential washes: **PBS** - **sodium-thiosulfate** - **PBS** for 2 min at 60 °C. Slides were stained at RT with **cresyl violet**, washed with **PBS**, dehydrated with a graded series of **alcohol** solutions and cleared with **xylene** and then cover slipped with **Entellan**.

### Kluver-Barrera staining

Sections of 5 μm were deparaffinised, dehydrated with 95% ethanol and immersed in **Luxol fast blue** solution at 56 °C overnight, after which the excess stain was rinsed off with (95%) **ethanol** followed by a rinse with distilled water. Differentiation of grey and white matters was performed by immersion in a **lithium carbonate** solution for 30 sec, followed by a (70%) **ethanol** rinse. Sections were washed with distilled water, counterstained with a **cresyl violet acetate** solution and mounted in a resinous medium. Image acquisition was performed using a Leica light microscope coupled to an ICC50 HD camera.

### Immunofluorescence

Entire brains were dehydrated with **sucrose** (15%) for 3 h and **sucrose** (30%) for 24 h, embedded in Tissue-Tek (OCT, Sakura) and sliced in a cryostat (Leica Biosystems) to obtain 20 μm coronal sections only of the cerebellum. Slices were hydrated with PBS for 5 min and immersed three times in PBST containing (0.5%) **Triton X-100** and (0.01%) T**ween 20** for 10 min; slices were washed twice with PBS for 5 min. Slices were incubated overnight at 4 °C with a **myelin basic protein** (MBP) primary **antibody AB40390** (Abcam) diluted (1:100) in PBS. Slices were immersed in an **anti-rabbit Alexa-594** secondary antibody diluted (1:1000) for 2 h and washed in PBS and PBST. Slices were contra-stained with **DAPI** (1:4,000 dilution) for 5 min and mounted in **Vectashield** (Vector) medium. The images were acquired using a Carl Zeiss 780 LSM confocal microscope. Confocal micrographs were acquired at 0.20 μm intervals of 1,024 × 1,024-pixel resolution and further processed and edited with the Image J (J64) and Amira (Amira 6.4.0 AmiraVR (XScreen) 6.2.4) software, respectively. Illustrative 3D video from Fig. 3 were obtained with the Amira software (Supplementary Video S6).

### Statistical analysis

Results were analysed using non-parametric Kruskal-Wallis tests coupled to a Dunn’s post-hoc test (control *vs*. treatments) with GraphPad Prism 7 software. Differences were considered statistically significant at *p* values < 0.05.

## Supplementary information

S1-S6
