# Supplementary material for: 3,5-T2 and 3,3′,5-T3 Regulate Cerebellar Thyroid Hormone Signalling and Myelin Molecular Dynamics in Tilapia
Source: Sci Rep. 2019 May 14;9:7359. doi: 10.1038/s41598-019-43701-w (PMC6517622; doi:10.1038/s41598-019-43701-w)
Supplement: Supplementary file 1 — S1-S6 [file 41598_2019_43701_MOESM1_ESM.pdf]

3,5-T2 and 3,3′,5-T3 Regulate Cerebellar Thyroid Hormone Signalling and Myelin Molecular Dynamics in Tilapia

Hernández-Linares Y., Olvera A., Villalobos P., Lozano-Flores C., Varela-Echavarría A., Luna M. and Orozco A.

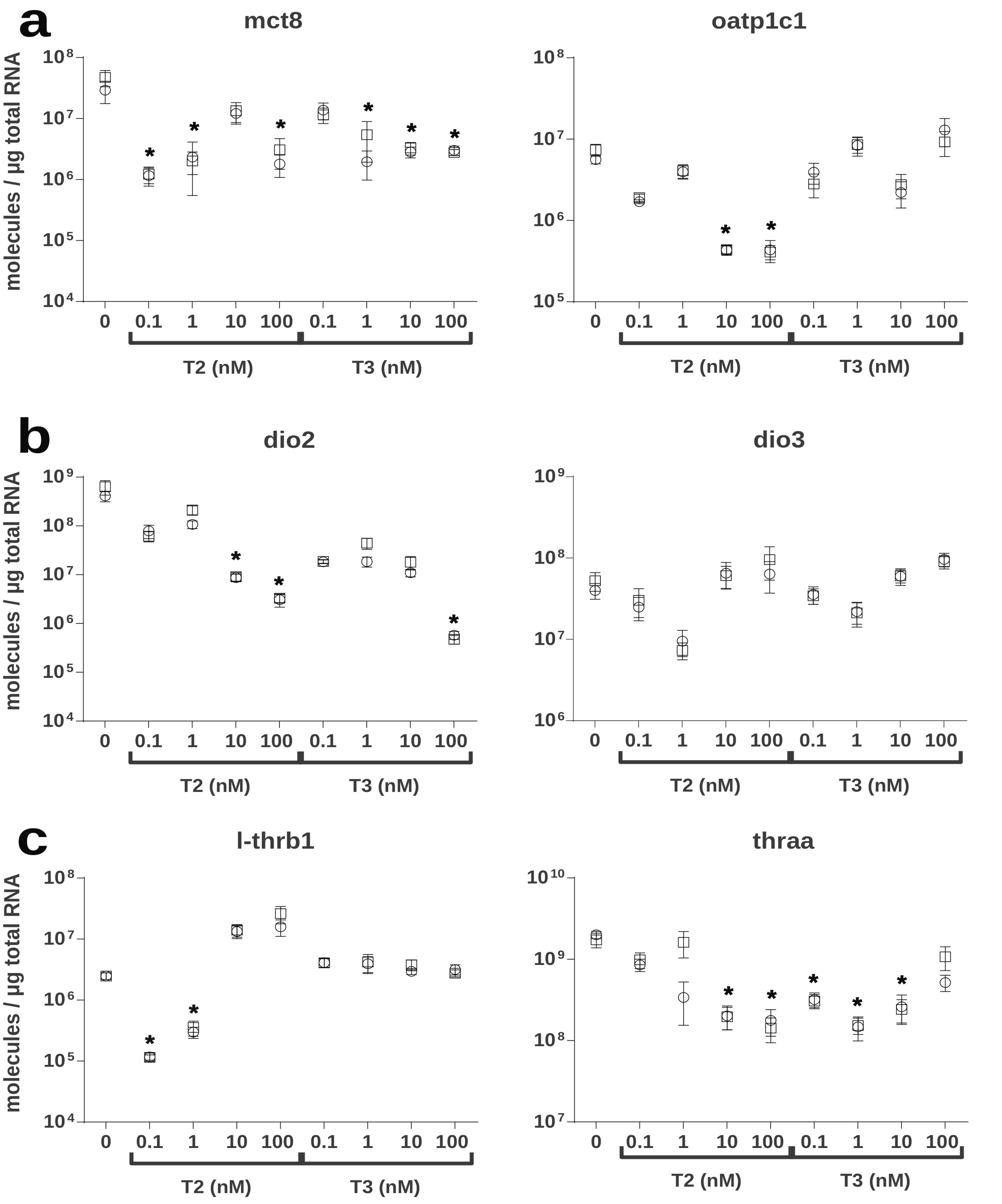

Supplemental Figure S1. Cerebellar mRNA expession of genes involved in thyroid hormone signalling. *Ex vivo* cultures of tilapia cerebella were treated with 0.01 N of NaCl (vehicle) in control groups or 0.1, 1, 10 or 100 nM of T2 and T3 for 24 h. Results shows qPCR normalised with two reference genes: ubiquitin-conjugating enzyme E2Z (ubce) in squares and beta actin (Actb) in circles. a) monocarboxilate transporter 8 (mct8) and organic anion transporting polypeptides type c1 (oatp1c1); b) deiodinase type 2 (dio2) and type 3 (dio3), and c) thyroid hormone receptors beta large (l-thrb1) and alfa (thraa). For all graphs * is $p < 0.001$

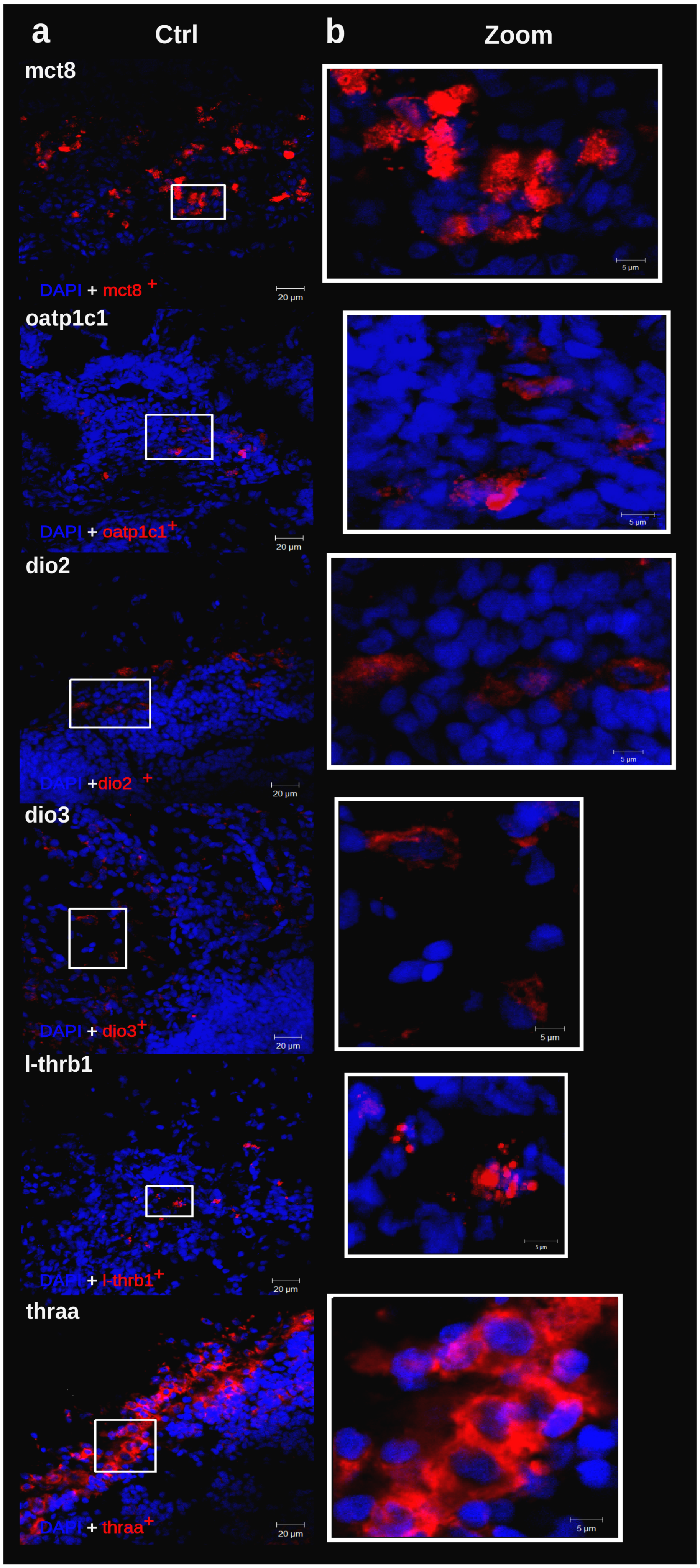

Supplemental Figure S2. Confocal images of mRNA expression of genes involved in thyroid hormone signaling. a) projection images acquired at 40x objective b) zooms of the zones of interest to show the cytoplasmic localisation of mRNA of each gene using FISH technique.

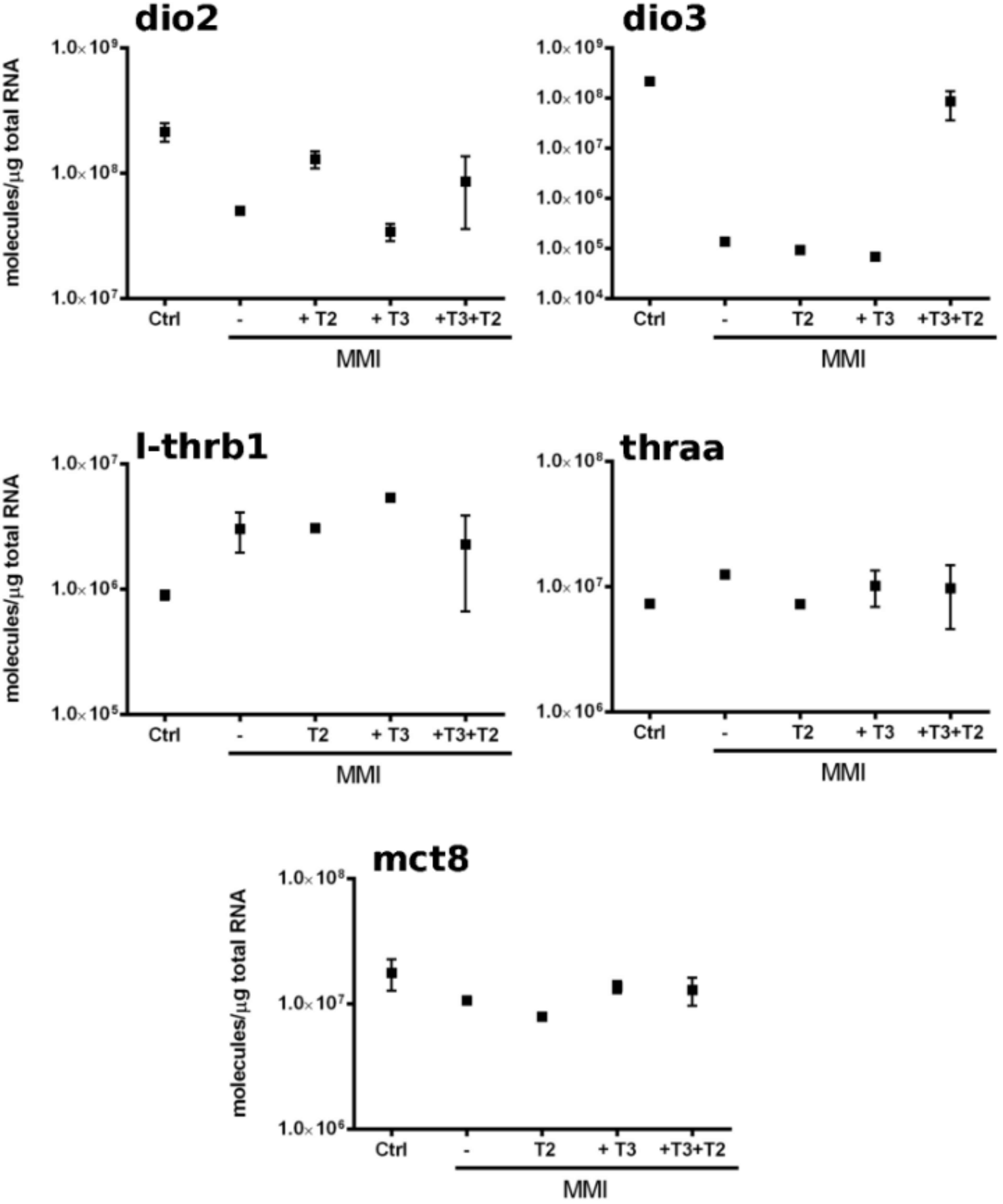

Supplementary Figure S3. mRNA expression of cerebellar dio2, dio3, l-thrb1, thraa and mct8. *In vivo* experiments, tilapia were expoused to 4.5 mM MMI with or without simultaneous addition of 1nM T2, T3 or T2+T3 for 30 days.
Values are means +/- S.E.M.

Name	abb	Reference	Primer F / R in 5'-3'	Amplicon (bp)	R Value
Thyroid hormone receptor alfa	thraa	AF302248.1	GCA GGA CTC TAA CCC ATC A<br>GTC ATG CTC TTC ACC GAA CA	96	0.97820
Thyroid hormone receptor beta long-isoform	l-thrb1	NM_001311334.1	GTG AAG GAA GCT AAG CCT GA<br>CAC AAG GCA GCT CAC AGA AC	172	0.95870
Deiodinase type II	dio2	XM_005477716.2	GAA ACT TGG CTG TGA GGC<br>TCA TCAATG TAC ACT AAC AGG	240	0.95450
Deiodinase type III	dio3	NM_001279439.2	GCA TCG CTG TTT GGAAGA CAG<br>TCT CAA AGT AGG CTC CGT ACG	125	0.93650
Solute carrier organic anion transporter family member 1c1	oatp1c1	XM_005451774.2	GAC CAC TGC TGG TTG TCA G<br>GTG TGATGG AGC TCT TCA TG	202	0.93890
Monocarboxilate transporter family 8	mct8	XM_005467565.2	GCT AAC GTT CAA GCC TCT GC<br>ACT CGG TAT GTG ACG ATG TG	180	0.98920
Actin citoplasmatic 2	Actb	XM_003455949	ACT TCG AGC AGG AGA TGG<br>GGT GGT TTC GTG GAT TCC	170	0.98970
Ubiquitin conjugating enzyme E2 Z	ube2z	XM_003460024	CTC TCAAAT CAA TGC CAC TTC C<br>CCC TGG TGG AGG TTC CTT GT	130	0.99360
Oligodendrocyte lineage transcription factor 2	olig2	ENSONIT00000021553.1	CCG TCA CCT CAG TCA GAC C<br>TGC AGG AGC TCT TTA GAG TC	205	0.9917
Transcription factor sox10	sox10	ENSONIT00000010558.1	GTG AAG AGG CCA ATG AAC GC<br>ATC TTC CCA TTC TTG CGG CG	239	0.9813
Myelin basic protein b	mbpb	ENSONIT00000009212.1	CAA CGC CAG TAG CAG AAC CT<br>TGAAGG TGT TGT CCT CTC GG	231	0.9936
Protein zero	p0	ENSONIT00000019027.1	GGC TCT GAC ATC CGA CTC TC<br>TGT CCA AAT AGG CAG CAC CA	145	0.9683
Proteolipid protein 1b	p1p1b	ENSONIT00000005464.1	AAG TGC TGT CAA GCA GAC CT<br>TGC TGA TTAATG CTG GCG GT	213	0.9898
Tankyrase 1	tnks	ENSONIT00000013634	AAG TCT GCT CTG GAT CTG GCT<br>CAT GTC CAC AGC GCT CTT GC	171	0.9889
Hepatic and glial cell adhesion molecule	glialCAM	ENSONIT00000014026	ACC TGC ATC AAC TCA GAG<br>ATT GGT GCC GCT CAT CCC GGT	169	0.9901

S4. Table 1, List of the oligonucleotides used for RT-qPCR and their principal characteristics.

Abbreviation	*Fragment size (bp)*	*Position*	*Linearization enzyme antisense/sense*
mct8	1131	307 - 1438	Not I – Spe I
oatp1c1	1207	1468 - 2675	Not I – Spe I
dio2	738	17 - 755	Not I – Pme I
dio3	801	129 - 930	Not I – Spe I
l-thrb1	1161	1 - 1161	Not I – Pme I
thraa	1133	58 - 1191	Not I – Spe I

S5. Table 2, List of the gene probes in pCR®4-TOPO® vector used for FISH and their principal characteristics.

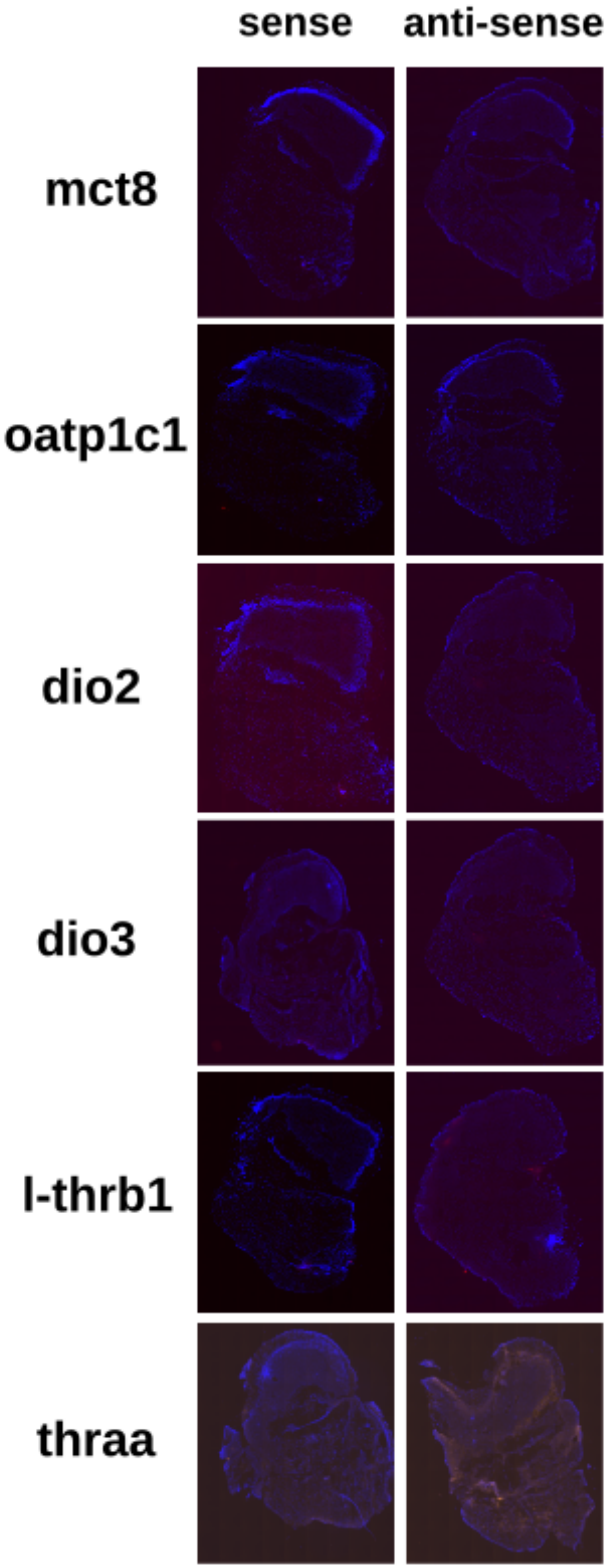

Supplemental Figure S6. Negative and positive controls for FISH. Sense probe (negative control) hybridization for each gene and anti-sense probe (positive) hybridization for each gene.

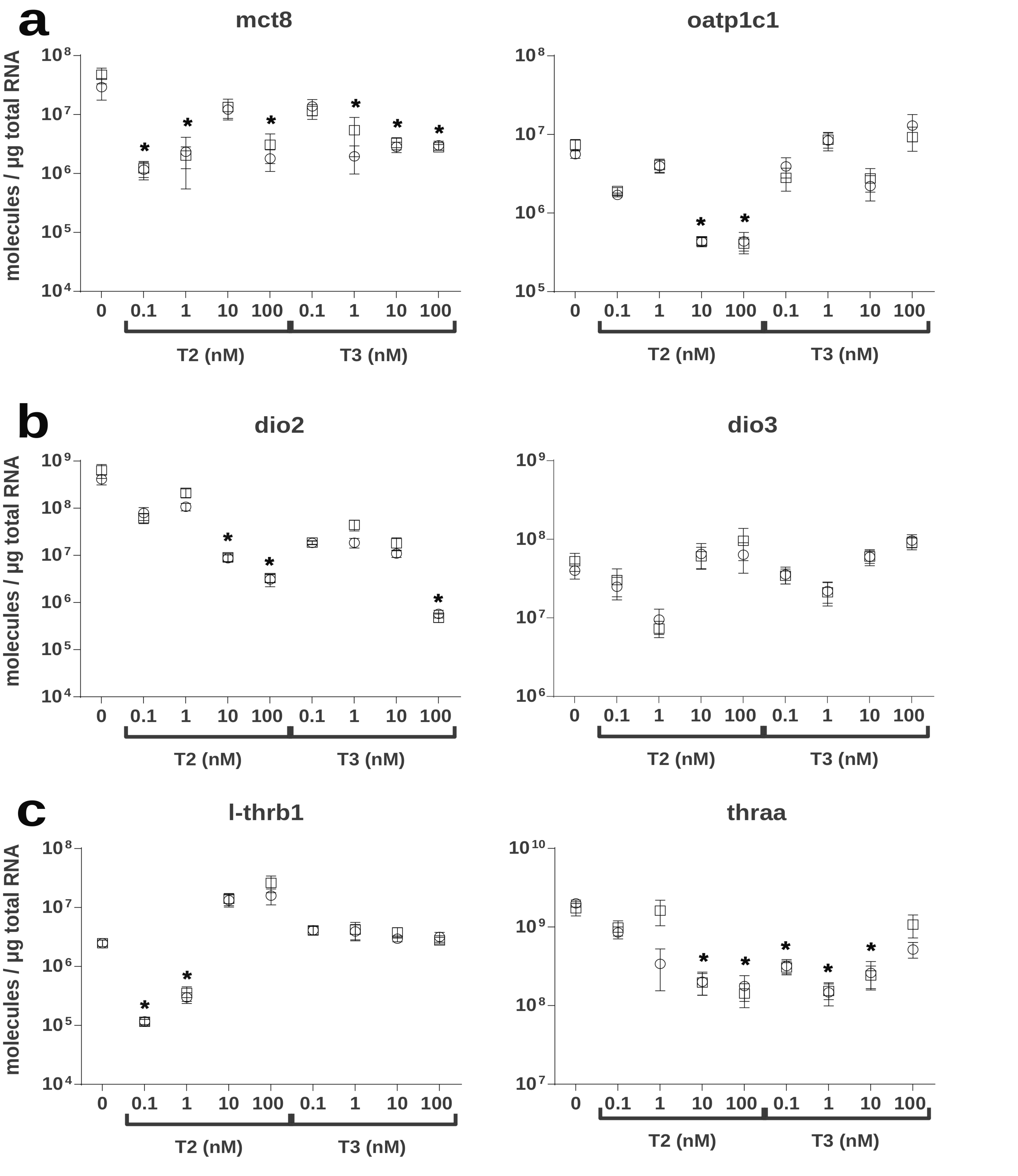

Supplemental Figure S1. Cerebellar mRNA expession of genes involved in thyroid hormone signalling. *Ex vivo* cultures of tilapia cerebella were treated with 0.01 N of NaCl (vehicle) in control groups or 0.1, 1, 10 or 100 nM of T2 and T3 for 24 h. Results shows qPCR normalised with two reference genes: ubiquitin-conjugating enzyme E2Z (ubce) in squares and beta actin (Actb) in circles. a) monocarboxilate transporter 8 (mct8) and organic anion transporting polypeptides type c1 (oatp1c1); b) deiodinase type 2 (dio2) and type 3 (dio3), and c) thyroid hormone receptors beta large (l-thrb1) and alfa (thraa). For all graphs * is $p < 0.001$

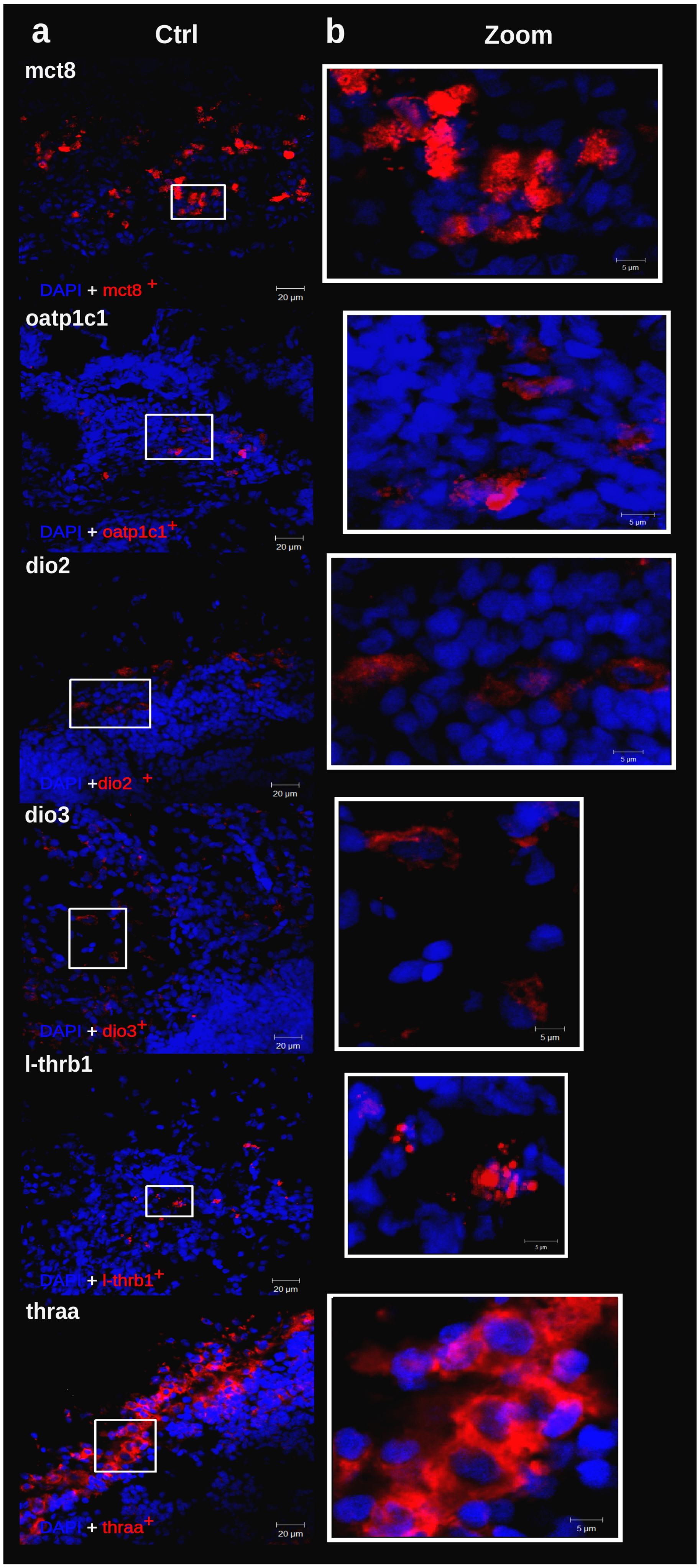

Supplemental Figure S2. Confocal images of mRNA expression of genes involved in thyroid hormone signaling. a) projection images acquired at 40x objective b) zooms of the zones of interest to show the cytoplasmic localisation of mRNA of each gene using FISH technique.

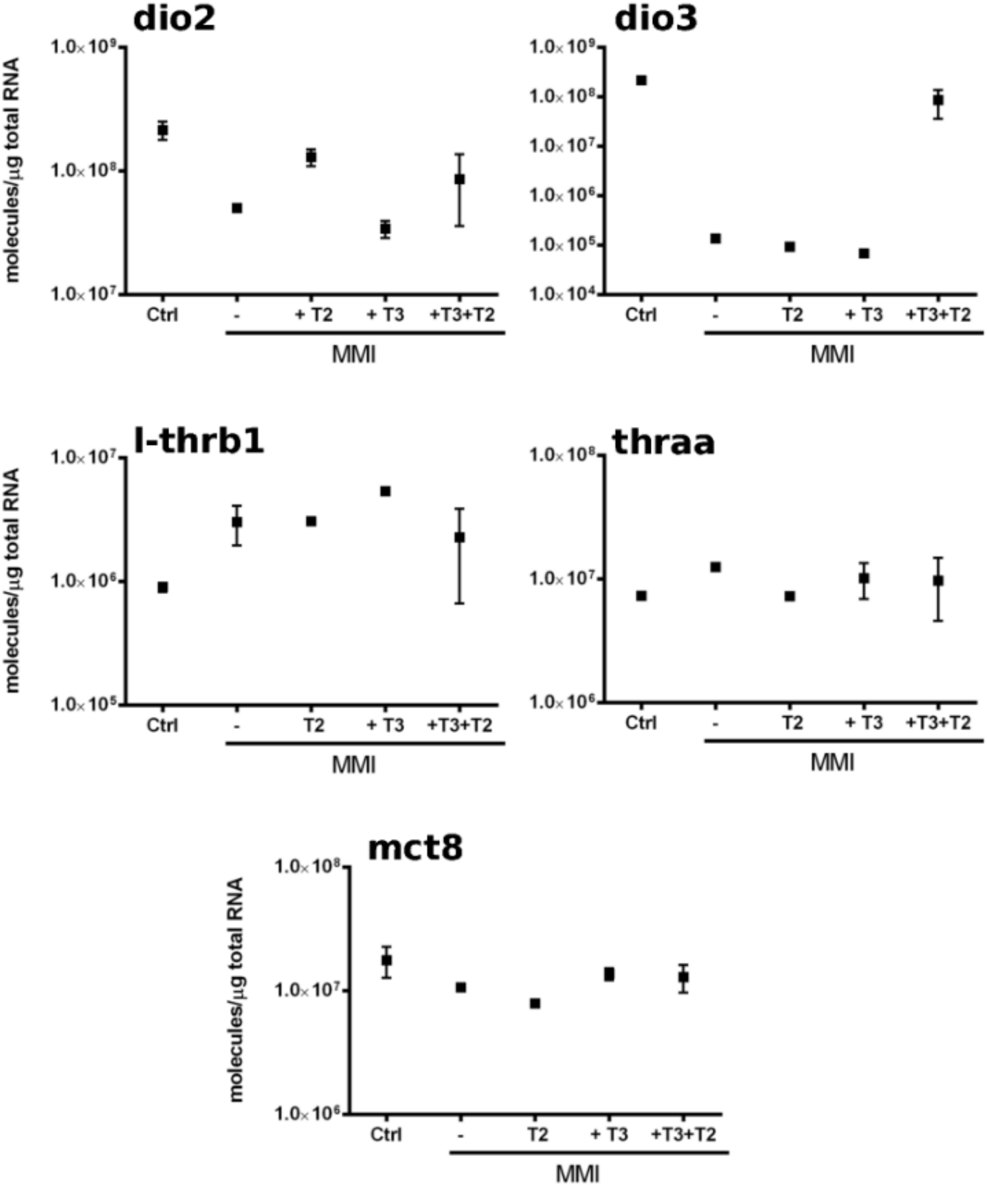

Supplementary Figure S3. mRNA expression of cerebellar dio2, dio3, l-thrb1, thraa and mct8. *In vivo* experiments, tilapia were expoused to 4.5 mM MMI with or without simultaneous addition of 1nM T2, T3 or T2+T3 for 30 days.
Values are means +/- S.E.M.

Table 1 S4. List of the oligonucleotides used for RT-qPCR and their main characteristics.

Name	abb	Reference	Primer F / R in 5'-3'	Amplicon (bp)	R Value
Thyroid hormone receptor alfa	thraa	AF302248.1	GCA GGA CTC TAA CCC ATC A<br>GTC ATG CTC TTC ACC GAA CA	96	0.97820
Thyroid hormone receptor beta long-isoform	l-thrb1	NM_001311334.1	GTG AAG GAA GCT AAG CCT GA<br>CAC AAG GCA GCT CAC AGA AC	172	0.95870
Deiodinase type II	dio2	XM_005477716.2	GAA ACT TGG CTG TGA GGC<br>TCA TCA ATG TAC ACT AAC AGG	240	0.95450
Deiodinase type III	dio3	NM_001279439.2	GCA TCG CTG TTT GGA AGA CAG<br>TCT CAA AGT AGG CTC CGT ACG	125	0.93650
Solute carrier organic anion transporter family member 1c1	oatp1c1	XM_005451774.2	GAC CAC TGC TGG TTG TCA G<br>GTG TGA TGG AGC TCT TCA TG	202	0.93890
Monocarboxilate transporter family 8	mct8	XM_005467565.2	GCT AAC GTT CAA GCC TCT GC<br>ACT CGG TAT GTG ACG ATG TG	180	0.98920
Actin citoplasmatic 2	Actb	XM_003455949	ACT TCG AGC AGG AGA TGG<br>GGT GGT TTC GTG GAT TCC	170	0.98970
Ubiquitin conjugating enzyme E2 Z	ube2z	XM_003460024	CTC TCA AAT CAA TGC CAC TTC C<br>CCC TGG TGG AGG TTC CTT GT	130	0.99360
Oligodendrocyte lineage transcription factor 2	olig2	ENSONIT00000021553.1	CCG TCA CCT CAG TCA GAC C<br>TGC AGG AGC TCT TTA GAG TC	205	0.9917
Transcription factor sox10	sox10	ENSONIT00000010558.1	GTG AAG AGG CCA ATG AAC GC<br>ATC TTC CCA TTC TTG CGG CG	239	0.9813
Myelin basic protein b	mbpb	ENSONIT00000009212.1	CAA CGC CAG TAG CAG AAC CT<br>TGA AGG TGT TGT CCT CTC GG	231	0.9936
Protein zero	p0	ENSONIT00000019027.1	GGC TCT GAC ATC CGA CTC TC<br>TGT CCA AAT AGG CAG CAC CA	145	0.9683
Proteolipid protein 1b	p1p1b	ENSONIT00000005464.1	AAG TGC TGT CAA GCA GAC CT<br>TGC TGA TTA ATG CTG GCG GT	213	0.9898
Tankyrase 1	tnks	ENSONIT00000013634	AAG TCT GCT CTG GAT CTG GCT<br>CAT GTC CAC AGC GCT CTT GC	171	0.9889
Hepatic and glial cell adhesion molecule	glialCAM	ENSONIT00000014026	ACC TGC ATC AAC TCA GAG<br>ATT GGT GCC GCT CAT CCC GGT	169	0.9901

Table 2 S5. List of the gene probes in pCR®4-TOPO® vector used for FISH and their principal characteristics.

Abbreviation	*Fragment size (bp)*	*Position*	*Linearization enzyme antisense/sense*
mct8	1131	307 - 1438	Not I – Spe I
oatp1c1	1207	1468 - 2675	Not I – Spe I
dio2	738	17 - 755	Not I – Pme I
dio3	801	129 - 930	Not I – Spe I
l-thrb1	1161	1 - 1161	Not I – Pme I
thraa	1133	58 - 1191	Not I – Spe I

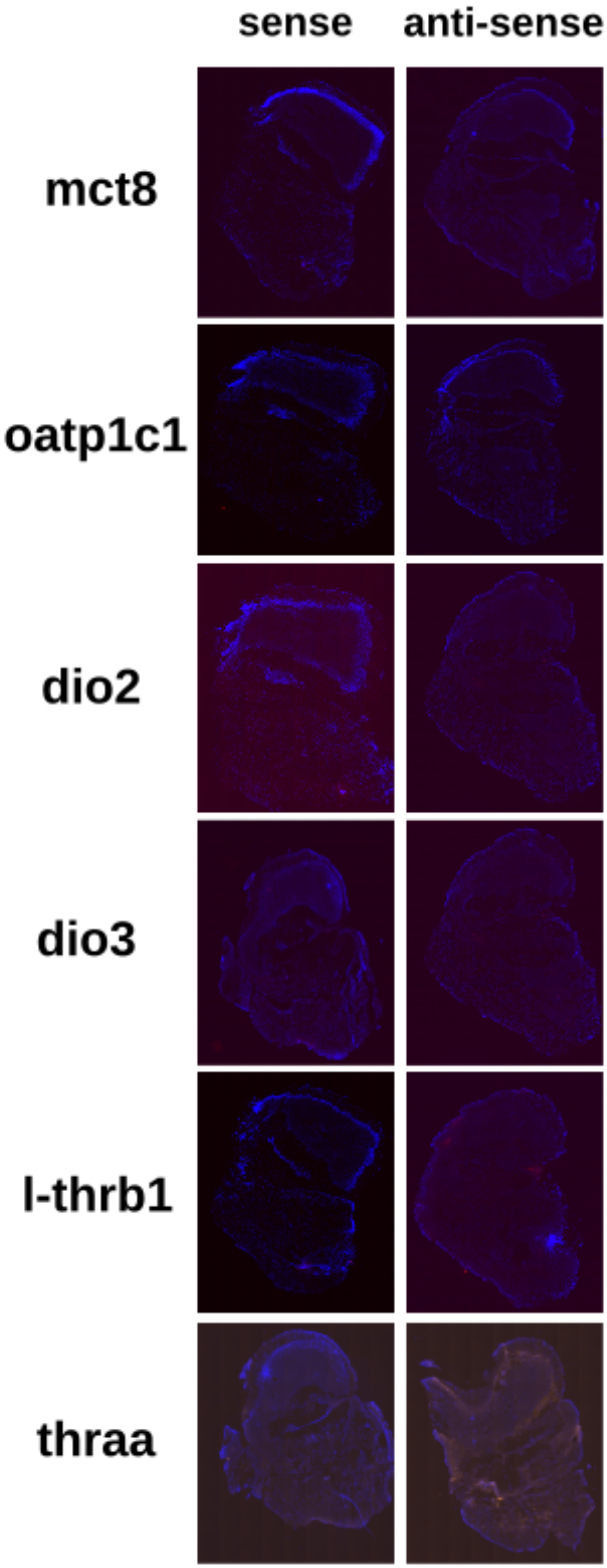

Supplemental Figure S6. Negative and positive controls for FISH. Sense probe (negative control) hybridization for each gene and anti-sense probe (positive) hybridization for each gene.
